# Simple Purification of *Nicotiana benthamiana*-Produced Recombinant Colicins: High-Yield Recovery of Purified Proteins with Minimum Alkaloid Content Supports the Suitability of the Host for Manufacturing Food Additives

**DOI:** 10.3390/ijms19010095

**Published:** 2017-12-29

**Authors:** Anett Stephan, Simone Hahn-Löbmann, Fred Rosche, Mirko Buchholz, Anatoli Giritch, Yuri Gleba

**Affiliations:** 1 Nomad Bioscience GmbH, Biozentrum Halle, D-06120 Halle (Saale), Germany; stephan@nomadbioscience.com (A.S.); hahn@nomadbioscience.com (S.H.-L.); gleba@nomadbioscience.com (Y.G.); 2 Fraunhofer Institute for Cell Therapy and Immunology, Department for Drug Design and Target Validation, D-06120 Halle (Saale), Germany; fred.rosche@izi.fraunhofer.de (F.R.); mirko.buchholz@izi.fraunhofer.de (M.B.)

**Keywords:** *Nicotiana benthamiana*, colicin, food additives, alkaloids, plant-made recombinant proteins, nicotine

## Abstract

Colicins are natural non-antibiotic bacterial proteins with a narrow spectrum but an extremely high antibacterial activity. These proteins are promising food additives for the control of major pathogenic Shiga toxin-producing *E. coli* serovars in meats and produce. In the USA, colicins produced in edible plants such as spinach and leafy beets have already been accepted by the U. S. Food and Drug Administration (FDA) and U. S. Department of Agriculture (USDA) as food-processing antibacterials through the GRAS (generally recognized as safe) regulatory review process. *Nicotiana benthamiana*, a wild relative of tobacco, *N. tabacum*, has become the preferred production host plant for manufacturing recombinant proteins—including biopharmaceuticals, vaccines, and biomaterials—but the purification procedures that have been employed thus far are highly complex and costly. We describe a simple and inexpensive purification method based on specific acidic extraction followed by one chromatography step. The method provides for a high recovery yield of purified colicins, as well as a drastic reduction of nicotine to levels that could enable the final products to be used on food. The described purification method allows production of the colicin products at a commercially viable cost of goods and might be broadly applicable to other cost-sensitive proteins.

## 1. Introduction

Colicins are natural non-antibiotic bacterial proteins with a narrow, usually species-specific, but highly potent antibacterial activity [1,2]. Colicins are produced by *Escherichia coli* and related bacteria. Recently, we have demonstrated that almost all known colicins can be expressed at high levels in green plants and that plant-made colicins are fully functional [3,4]. These proteins are being actively explored as promising candidates for controlling major *E. coli* pathovars. Notably, plant-made colicins have already been listed as GRAS (generally recognized as safe) by the U. S. Food and Drug Administration (FDA) and U. S. Department of Agriculture (USDA) and are allowed for application as *E. coli* antibacterials to a wide range of foods (FDA website: GRN 593 [5] and GRN 676 [6]). Colicin analogs from other Gram-negative bacteria (*Salmonella*, *Klebsiella*, *Pseudomonas*, etc.) are also being considered as non-antibiotic antibacterials for food safety and as edible and topical biopharmaceuticals [7,8,9].

*Nicotiana benthamiana*, a wild relative of tobacco, *N. tabacum*, has become the preferred production host plant for manufacturing recombinant proteins including biopharmaceuticals, vaccines, and biomaterials [2,10,11,12]. However, many members of the genus *Nicotiana* are known to contain high amounts of nicotine and, to a lesser extent, other related pyridine alkaloids in the green tissues of the plants [13,14]. Existing protocols for purifying antibodies, viral subunits, and virus-like particles (VLPs) from plants for clinical use results in highly pure proteins with low and acceptable residual nicotine levels, but are expensive and based on at least two or three chromatography steps [11,14]. We describe here a simple and inexpensive purification procedure for colicins transiently expressed in *N. benthamiana* that provides for high recovery efficiency of colicins—and perhaps other recombinant proteins intended to be used as food additives—and results in protein concentrates with a high content of colicins and very low levels of residual host alkaloids. The calculated maximum daily intake of nicotine based on the intended use of colicins as food antimicrobials when produced in *N. benthamiana* would be much lower than the actual nicotine intake from eating nicotine-containing foods such as tomatoes, potatoes, or eggplants, which are universally consumed safely as part of our standard diet.

## 2. Results

### 2.1. Development of a Purification Strategy for Colicins

**Figure 1:**
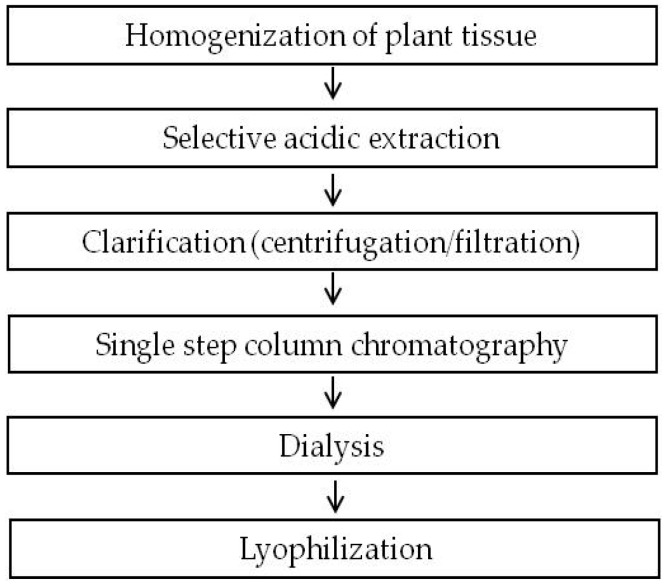
Flowchart of the experimental procedure for colicin purification from plant biomass by selective extraction, cation exchange chromatography, dialysis, and lyophilization.

Our previous study demonstrated broad and efficient control of foodborne pathogenic *E. coli* strains by different plant-made colicins [3]. In the current study, a simple and inexpensive purification strategy for the *N. benthamiana*-produced colicins ColM, ColK, ColU, and ColIb was established. These colicins were chosen over others because of their broader activity and higher potency against different pathogenic *E. coli* strains. The complementary mode of action and synergy of these colicins support their ultimate use as mixtures in food safety interventions. The goal of the study was to achieve an inexpensive purification of colicins, with a focus on improving protein purity and significantly reducing nicotine and other pyridine alkaloids in the resultant product while retaining high protein recovery yields. With these goals in mind, the selective acidic extraction of *N. benthamiana* biomass followed by a single chromatography step was evaluated (Figure 1).

**Figure 2:**
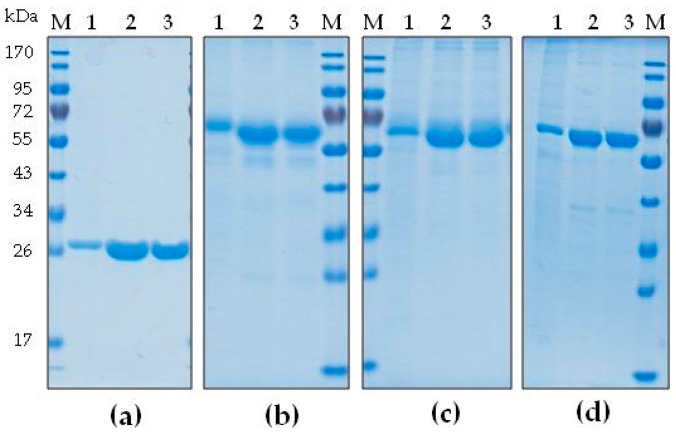
Batch purification of colicins M, K, U, and Ib. Samples were analyzed by sodium dodecyl sulfate polyacrylamide gel electrophoresis (SDS-PAGE) and Coomassie staining upon different steps of purification. Loading corresponds to 5 µL of each sample for ColM or 3.75 µL of each sample for ColK, ColU, and ColIb. (**a**) Purification of ColM; (**b**) Purification of ColK; (**c**) Purification of ColU; (**d**) Purification of ColIb. M: prestained protein ladder; 1: total soluble protein (TSP) extract; 2: colicin eluate (column purified); 3: purified colicin after dialysis.

**Figure 3:**
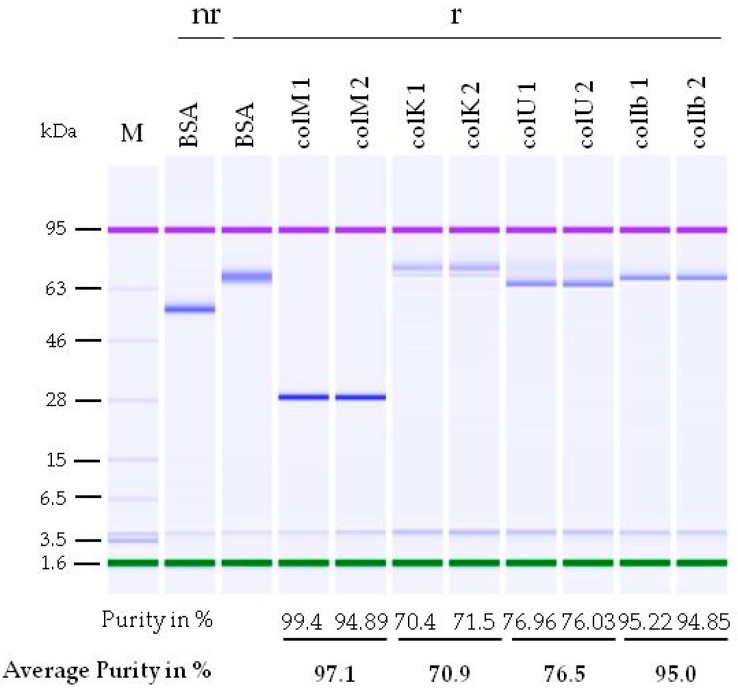
Capillary gel electrophoresis for evaluating the purity of the colicin preparations. One batch of lyophilized ColM, ColK, ColU, and ColIb was resuspended with H_2_O to a colicin concentration of 1 mg/mL and analyzed with an Agilent Protein 80 Kit (Agilent Technologies Deutschland, Waldbronn, Germany). The analysis was carried out in duplicate using bovine serum albumin (BSA) as a standard and the Agilent Protein 80 ladder (Agilent Technologies Deutschland, Waldbronn, Germany) (M). Proteins were separated under non-reducing (nr) and reducing (r) conditions. The purity of the colicin proteins is given as the percentage of total soluble protein obtained upon resuspension of the lyophilized colicin sample.

As shown by Schulz et al. [3], by using an acidic buffer (pH 4), colicins can be selectively extracted with an efficient reduction in host protein impurities in the total soluble protein (TSP) extract, especially the major leaf protein ribulose bisphosphate carboxylase. Therefore, this general approach was adapted for the purification of four colicins using pH 4.0–5.5, depending on the extraction efficiency of the colicins at this pH range. With the exception of ColK, the other colicins have a relatively high pI value (between 8.5 and 9); therefore, cation exchange chromatography was employed. As a column resin, CaptoMMC was used; it is a strong cation exchanger with a multimodal mode of action including hydrophobic interaction. One advantage of this resin is that the clarified plant extract can be loaded directly onto the equilibrated column, without any dilution to reduce conductivity. Subsequently, the eluted colicins were dialyzed against a buffer of neutral pH because previous studies have shown a high activity at pH 7.0 [3]. This strategy allowed the selected colicin proteins to be purified to 70–95% purity (Figure 2 and Figure 3).

Another advantage of this method is the very high recovery yield of the proteins during purification, as determined by sodium dodecyl sulfate polyacrylamide gel electrophoresis (SDS-PAGE) comparisons of the amount of colicin in the TSP extract (after acidic extraction) and in dialyzed samples relative to bovine serum albumin (BSA) as a reference. For ColM, ColK, ColU, and ColIb, the average protein recovery yields were 88%, 76%, 81%, and 55%, respectively (Table 1); it would be difficult to improve these further unless additional purification steps were included. The functionality of purified colicins was ensured by an antimicrobial activity test (Table 1) with no loss of specific antimicrobial activity being detected during the purification procedure. In analyses of batch-to-batch variation, the values of SD calculated for average specific colicin activity were greater than for recovery and yield of colicins, which is due to the less precise semi-quantitative method used. In part, the observed deviation rate also reflected the biological variation of the bacterial test strain employed for activity determination.

To ensure colicin integrity upon purification, reconstituted lyophilized colicins were analyzed by matrix-assisted laser desorption ionization—time of flight (MALDI-TOF/TOF) tandem mass spectrometry (Table 2). The molecular masses determined for the ionized colicins indicate that the proteins were intact and not truncated. Further proof of colicin integrity was found by N- and C-terminal protein sequencing by in-source decay (ISD). Table 2 shows that in general, ColM, ColK, and ColIb exhibit an N-terminal acetylation. For all colicins, the intact N- and C-terminus could be detected. ColM is the only colicin that exhibited an N-terminal methionine, which is in agreement with data from the literature [3,15]. In one batch of ColM, two other C-terminal degradation fragments were visible, which were underrepresented and could not be detected in the second batch analyzed.

For use as food antibacterials, the purity of colicin proteins can be lower than that for proteins typically used in medicine. For example, the commercially available antimicrobial protein nisin is sold as a powder consisting of only 2.5% nisin active ingredient; the balance being sodium chloride and denatured milk solids (Nisaplin^TM^, Danisco, Copenhagen, Denmark). Although the purity of the plant-made colicins already appeared quite high (Figure 2), one batch for each of the purified proteins was chosen for further analysis by capillary gel electrophoresis (Figure 3). The colicin batches were measured in duplicate and showed an average purity for ColM of 97%, ColK of 71%, ColU of 76.5%, and ColIb of 95%. The purity of ColIb as quantified by capillary gel electrophoresis (CGE) was higher than expected from the original analysis by SDS-PAGE (see Figure 2d). This difference could be the consequence of long-term storage of the lyophilized ColIb (7 months at 4 °C) between the time of SDS-PAGE and CGE analyses.

### 2.2. Determination of Alkaloid Content in Purified Colicin Samples

As mentioned, the purity of the colicins with respect to non-recombinant host protein impurities is not crucial for the application of colicins to food. Nevertheless, if the proteins are expressed in *N. benthamiana*, residual nicotine, anabasine, and other alkaloids in the final product should be reduced during purification to acceptable levels. According to Sisson et al. [15] who performed nicotine determination by gas chromatography (GC), green leaves of *N. benthamiana* contain on average 15.8 mg/g dry weight total alkaloids, most of it being nicotine (90.4%) and anabasine (8.4%) [16]. Accordingly, in our studies, we assumed that in wet leaves (90% moisture) we would find ~1.5 mg nicotine/g fresh weight plant material. Actual measurements of nicotine and anabasine (the most prominent pyridine alkaloids in *Nicotiana*) by high-performance liquid chromatography—tandem mass spectrometry (HPLC-MS/MS) showed amounts in the same range as those published (Table 3 and Table 4). Nevertheless, in our studies, we could show that the nicotine content is about 10-fold lower than published values. The same holds true in analyzing the anabasine content. This difference in alkaloid amount is most likely due to differences in experimental conditions (e.g., plant growth or extraction conditions).

We assumed that a level of 1000 ng nicotine per kilogram (kg) of food would be accepted as safe. According to Moldoveanu et al. [18], levels of nicotine in edible parts of tomato and eggplant are 3000–7000 ng/kg and according to Andersson et al. [19], average nicotine exposure from consumption of vegetables is approximately 1000 ng/day. We also calculated that a typical antibacterial application rate would be 6 mg of colicin per kilogram of meat. Therefore, the goal was to reduce the content of nicotine in colicin samples by at least 200-fold [18,19].

This study has clearly shown that nicotine and anabasine content can be drastically reduced after purification and dialysis (Table 5 and Table 6). After selective acidic extraction of the colicin-expressing plant material, nicotine levels ranged from 23,000 to 66,000 ng/mg protein. This amount could be reduced to 22–171 ng/mg protein (Table 5) using the purification procedure described. 

The initial levels of anabasine were 3200 to 8600 ng/mg protein (or 10-fold less in comparison to nicotine), which were reduced in most cases to just above the limit of detection (2.5 ng/mL; Table 6). In general, the highest nicotine and anabasine amounts were measured after purification of ColIb. This was not due to an inherently higher level of alkaloids in the sample because the values measured in TSP extracts between, for example, ColM and ColIb did not differ significantly. Rather, the discrepancy is most likely due to the lower protein concentration of ColIb after purification (Table 1).

In summary, our study has shown conclusively that the purification strategy employed reduced the nicotine and anabasine levels for ColM up to 1582 and 3323 times, respectively. The purification of ColK, ColU, and ColIb showed a reduction of nicotine by 2190, 1865, and 351-fold, respectively, with a corresponding reduction of anabasine by 920, 2016, and 195-fold.

## 3. Discussion

Currently, *N. benthamiana* is a preferred plant host for producing a wide range of recombinant proteins, for a number of reasons. First, production in a non-food species allows for clear segregation of the production host species and manufacturing process from the plants and processes intended to be used as foods or grown for other purposes. Second, the species has been extensively researched; its genetics, physiology, and agronomy are well understood [20,21], and it provides for very high recombinant protein expression levels when using either transient or transgenic expression. Multiple companies have built and/or are running GMP-compliant processes based on *N. benthamiana* as a production host; for example, Icon Genetics GmbH (Halle (Saale), Germany), Kentucky BioProcessing, Inc. (KBP) (Owensboro, KY, USA), iBio, Inc. (Newark, DE, USA), Medicago Inc. (Québec city, QC, Canada), and Fraunhofer USA (Newark, DE, USA). Although the plants yield less biomass per square meter than, for example, its commercial relative tobacco, *N. tabacum*, the recombinant protein yields are much higher; the net result is more recombinant protein per hectare with less biomass to process. Third, the safety of *N. benthamiana*’s use as a production host, as well as the products made in the species—including human vaccines, antibodies, and other proteins—have already been accepted by FDA, as evidenced by multiple product candidates in clinical evaluation under IND (Investigational New Drug) program [10,11,12,21]. To further reduce safety risks and improve product functionality, *N. benthamiana* production lines with humanized or designer glycosylation profiles have been engineered [22]. Although other crops such as spinach or leafy beets express colicins quite well, the upstream economics of *Nicotiana* are more favorable [23].

There are numerous well-established protocols for purifying biopharmaceutical proteins, vaccine antigens, and VLPs from tobacco, but those protocols all aim at very high protein purity levels and usually require at least two or three chromatography steps to achieve end results. Consequently, these procedures entail higher capital and operating costs [14,24]. Due to high product pricing, the production cost component for biopharmaceuticals and vaccines are less important; this is in sharp contrast to protein products to be used on food, where cost per treatment can be critical. There have also been attempts to purify tobacco soluble protein inexpensively with the goal of ultimately using said protein extract as food [25]. Attempts to monitor the amount of nicotine in the protein fractions and attempts to design a downstream process in such a way as to get rid of alkaloids have also been made [26]. We pursued a similar goal but our aim was to purify recombinant protein rather than the host’s soluble proteins, and thus our approach required an evaluation of numerous purification parameters including affinity resins and optimized buffer solutions. The protocol described here is robust, employs a single affinity chromatography step, and achieves high recovery, high yield (consequently reducing the purification cost), and up to 1500-times reduction in the levels of host alkaloids in the final product. We believe that the basic approach is generally applicable and that it can be used for purifying many cost-sensitive proteins, including those intended for use in food and feed, topical medicines, oral pharmaceuticals, etc.

The proposed downstream process described can provide a highly enriched recombinant protein fraction, constituting as high as 70–97% of the total soluble proteins. It is based on selective extraction, clarification using centrifugation and filtration, and just one chromatography step and employs a general-purpose resin (Capto MMC, GE Healthcare, Munich, Germany). Our results show that our process can reduce the nicotine content by 1000×–2000× in purified ColM, ColK, and ColU, and by 350× in purified ColIb. Purification of ColM, ColK, and ColU results in 900×–3500× reduction of the anabasine content, with a 200× reduction for ColIb purification. A future goal of our studies is to further reduce the amount of nicotine and anabasine in the purified ColIb preparations.

The commercial colicin blend and final use concentrations for control of Shiga toxin-producing *E. coli* (STEC) have been defined by Nomad as follows: Colicin M (3 mg/kg); Colicin Ib/a (1 mg/kg); Colicin K (1 mg/kg), and Colicin U (1 mg/kg); total 6 mg/kg (or 2.4 mg/lb). Our current purification process reduces the nicotine level in those colicins down to 30.75 ng/mg of dry protein powder for Colicin M, 150.15 ng/mg protein powder for Colicin Ib, 28.75 ng/mg protein powder for Colicin K, and 21.40 ng/mg protein powder for Colicin U. The total amount of nicotine in the defined blend would be 292.55 nanograms per 6 mg of the blend.

Daily meat (beef, pork, mutton, poultry) consumption per capita is 150 g for USA [27]. Consequently, the average expected U.S. daily dietary nicotine exposure from colicin-treated meat would be approximately 44 ng/day, which is a “worst case” scenario assuming that all meats are treated with the colicin product. That estimate will actually be much lower because it is unlikely that all consumed meat will be treated with colicins (i.e., less than 100% market penetration for the product). Based on publications summarized in Andersson et al. [19] and Moldoveanu et al. [18], the average daily nicotine exposure from consumption of solanaceous vegetables (mostly tomato, potato, and eggplant) is around 1000 ng/day. Our calculated daily exposure from consumption of meat treated with colicins would consequently be less than 5% of that from vegetables. It would also be approximately fifty thousand times less than a smoker’s exposure to nicotine from a single cigarette (2 mg nicotine/cigarette, [28]).

## 4. Materials and Methods

### 4.1. Bacterial Strains and Growth Conditions

*E. coli* DH10B cells were cultivated at 37 °C in LB medium (lysogeny broth). *Agrobacterium tumefaciens* ICF320 cells [14] were cultivated at 28 °C in LBS medium (modified LB medium containing 1% soya peptone; Duchefa, Haarlem, The Netherlands).

### 4.2. Plasmid Constructs

The coding sequence of Colicin Ib (GenBank entry: AAA23188.1) was codon-optimized by GeneArt for *N. benthamiana* and synthesized by Life Technologies (Darmstadt, Germany). ColIb sequence was cloned into a TMV-based magnICON destination vector using BsaI restriction sites. The ColM, ColK, and ColU constructs were described elsewhere [3].

### 4.3. N. benthamiana Plant Material and Protein Expression

*N. benthamiana* plants were grown in a greenhouse (day and night temperatures of 19 °C–23 °C and 17 °C–20 °C, respectively, with 12 h light and 35–70% humidity), and 6–7-week-old plants were used for inoculations. For plant transfection, saturated *Agrobacterium* overnight cultures were adjusted to OD_600_ 1.5 with *Agrobacterium* inoculation solution (10 mM 2-(*N*-morpholino)ethanesulfonic acid (MES), pH 5.5; 10 mM MgSO_4_) and were further diluted 1:100 in the same solution. The leaves were infiltrated using a needleless syringe. The infiltrated leaves were harvested at time points of maximum expression (ColIb = 5 days post infiltration (dpi), ColM = 6 dpi, ColK = 7 dpi, and ColU = 8 dpi) and plant material was ground in liquid nitrogen and stored at −80 °C. 

### 4.4. Protein Extraction and Purification

The number of purified protein batches for colicins M, K, U, and Ib was 9, 4, 8, and 13, respectively. In most cases, one batch was defined as an independently produced lot starting with the extraction of plant material and in some cases starting with a preparation of *Agrobacterium* cultures for infiltration of plant material.

For purification, plant material was extracted using the buffer containing 20 mM citric acid, 20 mM NaH_2_PO_4_, and 30 mM NaCl in a 5:1 (*v*/*w*) buffer:biomass ratio. The extraction was carried out at pH 4 in case of ColM and ColK and at pH 5.5 in case of ColIb and ColU. Ground leaf material supplemented with pre-chilled extraction buffer was incubated at room temperature under constant agitation for 30 min followed by centrifugation at 10,000× *g* for 15 min. The supernatant was filtered using Miracloth followed by incubation of the filtrate for 20 min at room temperature and centrifugation for 30 min at 10,000× *g* at 22 °C. The resulting supernatant was filtrated using filter discs with a pore size of 8–12 µm (TSP extract) and the final filtrate was used for further purification by cation exchange chromatography.

The filtrate was loaded on a CaptoMMC (GE Healthcare, Munich, Germany) column equilibrated with extraction buffer. The column was washed with extraction buffer to remove weakly bound non-target proteins. For further improvement of colicin purity, a second wash step was introduced using different amounts of elution buffer consisting of 50 mM Na_2_HPO_4_ (pH 7.84), 10 mM citric acid, and 1 M NaCl at a percentage adapted for each colicin as 10% for ColK and ColIb, 30% and 40% for ColM and ColU, respectively. The elution of ColM, ColK, and ColIb was carried out in a linear gradient until 100% of elution buffer concentration was reached over 6 column volumes. In the case of ColU, the elution was carried out in a step with 100% elution buffer. The eluted fractions were analyzed by SDS-PAGE and Coomassie staining using Instant Blue^TM^ staining solution (Expedeon, San Diego, CA, USA). The colicin containing fractions were pooled and dialyzed overnight at 4 °C against 20 mM Na_2_HPO_4_ (pH 7.5), 10 mM citric acid, and 50 mM NaCl. After dialysis, the proteins were frozen in liquid nitrogen and finally freeze-dried by lyophilization using freeze dryer Alpha 1–2 LDplus (Martin Christ Gefriertrocknungsanlagen, Osterode am Harz, Germany) for long-term storage of the colicins.

Protein concentrations were determined using Pierce^TM^ BCA Protein Assay Kit (Thermo Scientific, Waltham, MA, USA). The colicin recovery upon purification was determined by SDS-PAGE analysis and Coomassie staining by comparison of different amounts of TSP extract and dialyzed purified target proteins to a known amount of BSA standard. The percentage of recombinant colicins of TSP in plant extracts and colicin samples after purification was calculated based on total protein concentration and concentration of recombinant colicin estimated by SDS-PAGE-based visual comparison to BSA.

### 4.5. Colicin Antimicrobial Activity Determination

For semi-quantitative analysis of colicin activity during purification, growth inhibition tests on *E. coli* strain DH10B cells by spot on lawn soft agar overlay assays were carried out. For this, 5 µL of serial 1:0.5 dilutions of samples with 1% (*w*/*v*) milk powder (Carl Roth, Karlsruhe, Germany) were applied to an LB agar plate freshly seeded with soft agar at approximately 1 × 10^7^ cfu/mL (about 0.14 mL/cm²). The plates were incubated overnight at 37 °C. The number of colicin activity units (AU) per microliter (µL) was defined as the reciprocal of the highest dilution with a visible growth reduction effect on the DH10B cells. The specific colicin activity was calculated in the arbitrary units (AU) per microgram (µg) of protein.

### 4.6. Verification of Colicin Integrity

Protein samples were purified by solid-phase extraction using C_4_ or C_18_ bonded silica material (ZipTip^®^, Millipore, Darmstadt, Germany) and elution solutions were co-crystallized on a MALDI ground steel target with DHAP as well as DHB matrix (Bruker Daltonics, Bremen, Germany).

Mass spectra were acquired on a MALDI-TOF/TOF mass spectrometer (Autoflex Speed^TM^, Bruker Daltonics, Bremen, Germany) with positive polarity in linear mode for molecular mass determination and in reflector mode for ISD analysis. The matrix crystals were irradiated with a Nd:YAG laser (Smart beam-II^TM^, Bruker Daltonics, Bremen, Germany) at an emission wavelength of 355 nm and set to a pulse rate of 1 kHz. Spectra were recorded with flexControl (version 3.4, Bruker Daltonics, Bremen, Germany) by the accumulation of at least 10,000 laser shots per sample spot. Spectra processing was carried out with flexAnalysis (version 3.4, Bruker Daltonics, Bremen, Germany) by applying baseline subtraction with TopHat algorithm, smoothing with Savitzky–Golay algorithm and peak detection with SNAP algorithm. The mass spectrometer was calibrated using a set of standard peptides and proteins with known masses (Peptide Calibration Standard II, Protein Calibration Standards I and II, Bruker Daltonics, Bremen, Germany). 

Molecular mass determinations were based on the *m*/*z* values of single and multiple charged molecular ions. Annotation of ISD fragment spectra was carried out with the help of BioTools (version 3.2, Bruker Daltonics, Bremen, Germany) by in silico generation of *m*/*z* values for possible fragment ions and their comparison with the observed ISD fragment signals. This enabled the identification of the terminal amino acid sequences as well as of present posttranslational modifications. Both, molecular mass and ISD fragment signals were used to assess the integrity of the proteins.

### 4.7. Determination of Colicin Purity

For purity test of colicins, capillary gel electrophoresis (CGE)-on-a-chip analysis was performed on an Agilent 2100 bioanalyzer (Agilent Technologies Deutschland, Waldbronn, Germany) in combination with an Agilent Protein 80 Kit (sizing range: 5–80 kDa, Agilent Technologies Deutschland, Waldbronn, Germany) and 2100 Expert Software (Agilent Technologies Deutschland, Waldbronn, Germany)) [29]. All reagents and chips were prepared according to the manufacturer’s instructions.

Lyophilized, buffer-containing colicin samples were reconstituted with water to a concentration of 1 mg colicin per mL. Then, 4 µL of each colicin sample and 2 µL of reducing sample buffer were mixed and incubated at 95 °C for 5 min. After adding 84 µL water to each colicin-buffer mix, 6 µL of each sample was loaded onto a chip together with two BSA standard protein samples (reduced and non-reduced) and a Protein 80 ladder. The chip run results were displayed as a gel-like image, electropherograms, and in tabular form. Peak baseline adjusting and peak integration of electropherograms were done automatically and, if necessary, manual adjusting of peak baselines was done on a case-by-case basis.

### 4.8. Determination of Alkaloid Content

*N. benthamiana* plant leaf material was either homogenized by grinding in liquid nitrogen with subsequent extraction of total soluble protein (TSP) by supplementation with extraction buffer (20 mM Na-citrate, 20 mM Na_2_HPO_4_, 30 mM NaCl, pH 5.5) in 5:1 (*v*/*w*) buffer:biomass ratio or simultaneously homogenized and extracted with extraction buffer (2.5:1 (*v*/*w*) buffer:biomass ratio) in a lab blender (Memory blender, type RMBL, RotorLips AG). The supernatant of TSP extracts upon centrifugation for 20 min at 4500 rpm at room temperature was filtrated through Miracloth and analyzed by an HPLC-MS/MS-based method for alkaloids.

For calculation of alkaloid content per gram dry weight of *N. benthamiana*, the water content of the leaf material was determined by measurement of fresh weight and dry weight (upon drying of leaf material for 24 h at 80 °C followed by 48 h at 60 °C until no variation of weight was observed). For determination of alkaloid content in samples during colicin purification, TSP extracts or colicin-containing powders upon freeze-drying reconstituted with Millipore water were analyzed by HPLC-MS/MS-based method for alkaloids.

The HPLC-MS/MS-based quantification of the alkaloids nicotine and anabasine was done using an HPLC system (1260 Infinity, Agilent Technologies Deutschland, Waldbronn, Germany) coupled with a triple quad mass spectrometer (API3200; AB Sciex, Framingham, MA, USA). Nicotine and anabasine or nicotine-d4 and anabasine-d4 were separated by HPLC on a Synergi Max RP 2.5 µm, 50 × 2 mm (Phenomenex, Torrance, CA, USA) column with controlled temperature at 60 °C and subsequently detected by tandem mass spectrometry (MS/MS) using an API ion source (TurboIon Spray™, AB Sciex, Framingham, MA, USA). Standard solutions of nicotine, anabasine, and the corresponding deuterated d4 derivatives in methanol were purchased from Sigma-Aldrich (Hamburg, Germany). All solvents used were of ULC-MS grade and purchased from Biosolve (Valkenswaard, Netherlands). 

For the quantification of nicotine, a solution of 50 ng/mL nicotine-d4 in 90% acetonitrile containing 0.1% formic acid was used as the internal standard. Samples consisting of 100 μL of 50 ng/mL nicotine-d4, 50 μL 90% acetonitrile, 0.1% formic acid, and 50 μL sample (plant TSP extract or reconstituted freeze-dried purified colicin powder) or diluted sample were mixed. Samples consisting of 100 μL 50 ng/mL nicotine-d4 solution, 50 μL nicotine in 90% acetonitrile, 0.1% formic acid at different concentrations (5, 10, 20, 50, 100, 500, 1000, 1500 ng/mL), and 50 μL water served as the standard. Samples and standards were incubated at 4 °C for 10 min and centrifuged at 4 °C at 24,000× *g* for 10 min. The filtrated supernatant was diluted 1:5 in water and transferred into autosampler vials. For the HPLC system, the following parameters were used: 5 µL injection volume, mobile phases: [A] water (5 mM ammonium formate) and [B] acetonitrile (5 mM ammonium formate) applied in a gradient at a flow rate of 0.4 mL/min and a total analysis time of 7.50 min: during run time from 0–5.00 min, [A] and [B] were applied from 98% to 5% and 2–95%, respectively, followed by [A] and [B] from 5% to 98% and 95% to 2%, respectively, during run time from 5.00–7.50 min. The analyte and internal standard were detected at 2.00 min. The limit of quantitation was reached at a concentration of 5 ng/mL and linearity was obtained for concentrations ranging from 5 ng/mL to 1500 ng/mL. The multiple reaction monitoring (MRM) signals were analyzed for nicotine-1 (Q1 mass of 163.3 Da and Q3 mass of 132.1 Da (quantifier)), nicotine-2 (Q1 mass of 163.3 Da and Q3 mass of 106.0 Da (qualifier)), nicotine-d4-1 (Q1 mass of 167.1 Da and Q3 mass of 134.2 Da (quantifier)), and nicotine-d4-2 (Q1 mass of 167.10 Da and Q3 mass of 121.1 Da (qualifier)), whereas for each MRM the ion conditions were optimized separately.

For the quantification of anabasine, a solution of anabasine-d4 in 90% acetonitrile containing 0.1% formic acid was used as the internal standard. Anabasine in 90% acetonitrile and 0.1% formic acid at different concentrations (2.5, 5.0, 10, 50, 100, 200, 500 ng/mL) was used as a standard. Sample preparation and processing by HPLC was done as described above for nicotine quantification. The analyte and internal standard were detected at 1.90 min. The limit of quantitation was reached at a concentration of 2.5 ng/mL and linearity was obtained for concentrations ranging from 2.5 ng/mL to 500 ng/mL. The MRM signals were analyzed for anabasine-1 (Q1 mass of 163.2 Da and Q3 mass of 92.0 Da (quantifier)), anabasine-2 (Q1 mass of 163.2 Da and Q3 mass of 14.2 Da (qualifier)), anabasine-d4-1 (Q1 mass of 167.1 Da and Q3 mass of 96.1 Da (quantifier)), and anabasine-d4-2 (Q1 mass of 167.10 Da and Q3 mass of 122.1 Da (qualifier)), whereas for each MRM the ion conditions were optimized separately.

For data evaluation, the peak areas obtained for the respective alkaloid in TSP extract or purified colicin samples and standard samples were normalized by the peak areas obtained for the internal standard spiked into each sample. The data were recorded, edited, and evaluated using the Analyst™ 1.6.2 software package (AB Sciex, Framingham, MA, USA). Quality control samples prepared by supplementation of selected original samples with standard were analyzed in every batch. A deviation of 15% between the theoretical and calculated concentration was specified.

## Figures and Tables

**Table 1:** Analysis of different batches of purified colicins for the recovery of active antimicrobial proteins. Lyophilized protein samples were inspected for yield of recombinant colicins and yield in specific colicin antimicrobial activity. Antimicrobial activity is expressed in AU (arbitrary units) per microgram (µg) recombinant colicin. Average numbers were calculated per batch based on numbers of batches for each colicin as indicated.

Colicin	Number of Batches	Average Fresh Weight Plant Material (g)	Average Recovery of Colicin upon Purification (%)	Average Specific Activity (AU/µg Colicin)	Average Yield of Lyophilized Colicin (mg)
ColM	9	54	88 ± 6.1	2.47 × 10^6^ ± 2.11 × 10^6^	72.6 ± 17.8
ColK	4	50.7	76 ± 8.9	1.89 × 10^6^ ± 7.56 × 10^5^	117.3 ± 19.9
ColU	8	58.8	81 ± 10	1.08 × 10^6^ ± 9.34 × 10^5^	81.6 ± 16.4
ColIb	13	59.7	55 ± 9.9	3.19 × 10^6^ ± 2.67 × 10^6^	35.2 ± 14

**Table 2:** Mass spectrometry—based analysis of the integrity of purified colicins by determination of the molecular mass and verification of the N- and C-terminal sequences by in-source decay measurements.

Protein	Proteoform	Mass (Da)	Amino Acid Sequence and Posttranslational Modification	
			N-Terminus	C-Terminus
ColM Batch 1	1	29,519.7	Acetyl-METLTVHAPS(…)	(…)GEIHIKESGKR-OH
	2	29,088.2	Acetyl-METLTVHAPS(…)	(…)GEIHIKE-OH
	3	28,963.2	Acetyl-METLTVHAPS(…)	(…)GEIHIK-OH
ColM Batch 2	1	29,521.0	Acetyl-METLTVHAPS(…)	(…)GEIHIKESGKR-OH
ColK	1	59,569.7	Acetyl-AKELSGYGP(…)	(…)SKLNELLGI-OH
ColU	1	66,163.9	H_2_N-PGFNYGGHG(…)	(…)LNNEIIRPAY-OH
ColIb	1	69,784.7	Acetyl-SDPVRITNPG(…)	(…)IEQVNKLIGI-OH
	2		H_2_N-SDPVRITNPG(…)	(…)IEQVNKLIGI-OH

**Table 3:** Nicotine content of non-treated *N. benthamiana.* Total soluble protein (TSP) extracts analyzed for alkaloid content by high-performance liquid chromatography—tandem mass spectrometry (HPLC-MS/MS) were prepared with two different extraction methods and the nicotine contents determined were compared to literature.

Sample	Nicotine Content ng/g Fresh Weight		Nicotine Content ng/g Dry Weight	
	Average	Literature	Average	Literature
*N. benthamiana* leaf, ground in N_2_^1^	123,667 ± 59,181	~500,000 [17]	690,000 ± 340,000	~14,000,000 [16], ~3,000,000 [13]
*N. benthamiana* leaf, extracted with lab blender ^1^	103,330 ± 15,610		1,140,000 ± 190,000	

^1^ Buffer consisting of 20 mM citrate, 20 mM Na_2_HPO_4_, 30 mM NaCl (pH 5.5) was used for the extraction. Samples were analyzed in triplicate.

**Table 4:** Anabasine content of non-treated *N. benthamiana.* TSP extracts analyzed for alkaloid content by HPLC-MS/MS were prepared with two different extraction methods and the nicotine contents determined were compared to literature.

Sample	Anabasine Content ng/g Fresh Weight		Anabasine Content ng/g Dry Weight	
	Average	Literature	Average	Literature
*N. benthamiana* leaf, ground in N_2_^1^	14,133 ± 2590	no literature about anabasine content per g fresh weight biomass available	80,000 ± 20,000	~1,300,000 [16] (9.3% of nicotine)
*N. benthamiana* leaf, extracted with lab blender ^1^	12,930 ± 400		140,000 ± 10,000	

^1^ Buffer consisting of 20 mM citrate, 20 mM Na_2_HPO_4_, 30 mM NaCl (pH 5.5) was used for the extraction. Samples were analyzed in triplicate.

**Table 5:** Nicotine content of colicin-containing samples during and after purification. Colicin-containing samples of two batches were analyzed by HPLC-MS/MS for quantification of nicotine. Analysis was carried out for two batches of each colicin.

Colicin	Nicotine Concentration (ng/mg TSP)		
	Extraction	Purification and Dialysis	Lyophilization
ColM	48,654 ± 1295	18.32 ± 4.66	30.75 ± 12.76
ColK	62,988 ± 4417	19.64 ± 15.59	28.75 ± 21.45
ColU	39,905 ± 23,750	33.07 ± 29.76	21.41 ± 2.4
ColIb	52,665 ± 6819	137.3 ± 11.64	150.15 ± 30.67

**Table 6:** Anabasine content of colicin-containing samples during and after purification. Colicin-containing samples were analyzed by HPLC-MS/MS for quantification of anabasine. The analysis was carried out for two batches of each colicin.

Colicin	Batch	Anabasine Concentration (ng/mg TSP)		
		Extraction	Purification and Dialysis	Lyophilization
ColM	1	7404	<1.21	<1.54
	2	4095	2.83	<1.92
ColK	1	7644	<1.75	13.91
	2	7047	2.44	2.06
ColU	1	3178	<2.34	<2.5
	2	7647	3.67	2.87
ColIb	1	7569	23.61	43.59
	2	8637	39.26	39.18

## Abbreviations

DHAP	2,5-dihydroxyacetophenone
DHB	2,5-dihydroxybenzoic acid
ISD	in-source decay
*m*/*z*	mass-to-charge ratio
